# Tacrolimus-induced Ascites after Liver Transplant

**Published:** 2018-05-01

**Authors:** M. Hosseini, M. Aliakbarian, K. Akhavan-Rezayat, O. Shadkam, S. Milani

**Affiliations:** 1 Department of Internal Medicine, Faculty of Medicine, Mashhad University of Medical Sciences, Mashhad, Iran; 2 Surgical Oncology Research Center, Mashhad University of Medical Sciences, Mashhad, Iran

**Keywords:** Ascites, Tacrolimus, Liver transplantation, Immunosuppressive agents

## Abstract

Massive post-transplantation ascites is a rare but serious condition following liver transplantation. Although, many etiologies are suggested as the cause of this complication, in some cases the definitive etiology remains unknown. Drug-induced post-transplantation ascites is one of the possible etiologies. In this study we present a case of ascites caused by tacrolimus in the post-liver transplantation period. A 49-year-old man with hepatitis B virus cirrhosis underwent liver transplantation and received tacrolimus, mycophenolate and prednisolone, as the immunosuppressive regimen. Progressive ascites developed after 10 days, in spite of a normal liver function. Various studies, including liver biopsy, were performed but we could not find any etiology for this complication. The tacrolimus was switched to rapamune. Ascites was completely disappeared and up to the last follow-up visit, the patient remained asymptomatic for more than two years. We concluded that after ruling out other etiologies, tacrolimus as a rare cause of post-transplantation ascites should be taken into account. The treatment is discontinuation of the drug.

## INTRODUCTION

Ascites is the collection of fluid in the space between the lining of the abdomen and abdominal organs. It results from different etiologies such as portal hypertension, hypoalbuminemia, infection, malignancy, liver disease, heart failure, and peritoneal inflammation [1]. Ascites is one of the most common complications after liver transplantation, which is usually transient and self-limited [2]. Conversely, massive and refractory ascites are seen infrequently (5%–7%) in liver transplant recipients, however, it may cause morbidity and mortality [3]. There are some other less-known or even unknown causes of ascites after liver transplantation such as tacrolimus therapy. Studies showed that liver dysfunction can impair the metabolism of tacrolimus, frequently reported in liver transplant recipients [4]. 

Tacrolimus, as a calcineurin inhibitor derived from a soil fungus, Streptomyces tsukubaensis, is one of the mainstays in immunosuppressive regimens [5]. This agent binds to calcineurin that can block transcription of factors IL2, IL3, IL4, IL5, CD40 ligand, GM-CSF, IFNγ, and TNFα. Furthermore, it can stop cytokine receptor expression and cytokine effects on target cells [6]. The major drawbacks of tacrolimus are its narrow therapeutic window, unpredictable bioavailability, and its association with development of post-transplantation diabetes mellitus, and nephrotoxicity [7]. Fluid and sodium retention are reported too [8]. For patients with tacrolimus-related adverse effects, a sirolimus-based immunosuppression regimen is a rescue therapy [9].

Sirolimus (Rapamycin), as an immunosuppressive drug belonging to the target-of-rapamycin (TOR) inhibitors family, is used in prevention of organ rejection after transplantation [10]. It inhibits mammalian target of Rapamycin (mTOR) and blocks the activation of IL2 and inhibits the progression of the T cell from the G phase to the S phase and also affects IL4, IL7, IL12, and IL15 [11].

Herein, we present a case of ascites following liver transplantation who had been treated with tacrolimus, and who achieved complete clinical remission after conversion of tacrolimus to sirolimus.

## CASE PRESENTATION

A 49-year-old Iranian man underwent liver transplantation for chronic hepatic failure due to hepatitis B and cirrhosis (MELD score of 20). Early post-operative recovery period was uneventful. An immunosuppressive regimen comprising prednisone, tacrolimus and cellcept were administered. Trough blood concentration of tacrolimus was maintained at 7–8.7 ng/mL with the daily dose of 4 mg. Hepatic function and coagulation status were within normal ranges 15 days after the transplantation. 

The patient did not experience any serious complications and clinical episodes except for ascites on the 10^th^ postoperative day. Ultrasonographic evaluation showed massive ascites. Color Doppler ultrasonography of the hepatic vessels, inferior vena cava and portal vein demonstrated normal findings. Ascites was resistant to treatment and the weekly therapeutic aspiration of the ascitic fluid was necessary to alleviate symptoms. Cytological, biochemical and microbiological analyses of the ascitic fluid had unremarkable results. A liver biopsy was obtained under ultrasound guidance, which showed no significant abnormality in two separate pathological reviews. Cytomegalovirus viral load was analyzed, which presented a level below the lower limit of the assay (700 copies/mL) in plasma.

Tacrolimus concentration was analyzed. Tacrolimus was considered as the only offending drug that was subsequently withdrawn and substituted by sirolimus at a concentration of 5 ng/mL. Resolution of ascites was observed 10 days after conversion of tacrolimus to sirolimus. In his last follow-up visit, the patient has remained asymptomatic for more than two years.

## DISCUSSION

Medical and surgical complications including graft rejection, bacterial infections, and vascular or biliary problems are seen frequently after liver transplantation. The development of ascites after transplantation is often detected in the early post-operative stage, which usually disappears in a few days. On the other hand, massive and prolonged ascites are observed in some cases, which can progress toward serious adverse events. In this condition, patients need to be managed for diminishing the risks of renal failure development and requiring a lengthy hospital stay. These cases have higher risk of intraperitoneal infections, electrolyte abnormalities, and, in severe cases, even graft loss and death. 

Stenosis of the inferior caval vein anastomosis [12], decreased liver vascular compliance during acute cellular rejection [13], and the use of reduced grafts causing inadequate accommodation of liver blood flow [14] have been reported as the possible causes of post-operative ascites. Although, some probable causes, which can play a role in the pathogenesis of massive post-liver transplantation ascites, may remain unknown. One of the rare possible etiologies is drug-induced ascites. The use of tacrolimus in preventing graft rejection following organ transplantations is associated with both risks and benefits. 

Some theories are suggested possible mechanisms for drug-induced ascites following tacrolimus administration. In contrast to the rapid improvement of liver function in response to the higher doses of tacrolimus, this episode may trigger intractable ascites [15]. Since the ascites after liver transplantation can be associated with marked renal impairment [2], nephrotoxicity may be regarded as the cause of ascites. As well, the studies have shown that tacrolimus is involved in the development of sinusoidal obstruction syndrome (SOS) after liver, pancreas, and lung transplantation [16]. Metabolism of tacrolimus is more severely affected in patients with hepatic dysfunction, especially in SOS rather than other etiologies, which in turn, may result in prolonged half-life and reduced clearance of tacrolimus [17]. Regarding the development of ascites as one of the symptoms of SOS after liver transplantation, it can be attributed to tacrolimus composition and impairment of its metabolism. 

Fluid retention presenting as ascites, edema, or pleural effusion may occur in 15%–35% of patients receiving tacrolimus [18], which may be due to drug-induced serosal inflammation. In our case, all possible etiologies for post-transplantation ascites were studied and ruled out. Absence of other factors and resolution of the ascites following discontinuation of tacrolimus proposed serosal inflammation as the likely underlying mechanism for the ascites.

In conclusion, in the event of post-liver transplantation ascites, tacrolimus consumption, as a probable cause, must always be kept in mind after ruling out other etiologies.

## References

[1] Reynolds TB (2000). Ascites. Clinics in Liver Disease.

[2] Kitajima K, Vaillant JC, Charlotte F (2010). Intractable ascites without mechanical vascular obstruction after orthotopic liver transplantation: etiology and clinical outcome of sinusoidal obstruction syndrome. Clin transplant.

[3] Cirera I, Navasa M, Rimola A (2000). Ascites after liver transplantation. Liver Transplantation.

[4] Mori T, Shimizu T, Yamazaki R (2005). Altered metabolism of tacrolimus in hepatic veno-occlusive disease. Transplant International.

[5] Wallemacq PE, Reding R (1993). FK506 (tacrolimus), a novel immunosuppressant in organ transplantation: clinical, biomedical, and analytical aspects. Clin Chemis.

[6] Rostaing L, Puyoo O, Tkaczuk J (1999). Differences in Type 1 and Type 2 intracytoplasmic cytokines detected by flow cytometry, according to immunosuppression (cyclosporine A vs tacrolimus) in stable renal allograft recipients. Clin transplant.

[7] Scholten EM, Cremers SCLM, Schoemaker RC (2005). AUC-guided dosing of tacrolimus prevents progressive systemic overexposure in renal transplant recipients. Kidney Int.

[8] Hoorn EJ, Walsh SB, McCormick JA (2011). The calcineurin inhibitor tacrolimus activates the renal sodium chloride cotransporter to cause hypertension. Nat Med.

[9] Yu S, He X, Yang L (2008). A retrospective study of conversion from tacrolimus-based to sirolimus-based immunosuppression in orthotopic liver transplant recipients. Exp Clin Transplant.

[10] Trotter JF (2003). Sirolimus in liver transplantation. Transplant Proc.

[11] Dumont FJ, Su Q (1995). Mechanism of action of the immunosuppressant rapamycin. Life Sciences.

[12] Zajko AB, Claus D, Clapuyt P (1989). Obstruction to hepatic venous drainage after liver transplantation: treatment with balloon angioplasty. Radiology.

[13] Mabrut JY, de la Roche E, Adham M (1998). Peritoneovenous diversion using the LeVeen shunt in the treatment of refractory ascites after liver transplantation. Annales de chirurgie.

[14] Adetiloye VA, John PR (1998). Intervention for pleural effusions and ascites following liver transplantation. Ped radiology.

[15] Kitajima K, Vaillant J-C, Charlotte F (2010). Intractable ascites without mechanical vascular obstruction after orthotopic liver transplantation: etiology and clinical outcome of sinusoidal obstruction syndrome. Clin transplant.

[16] Shen T, Feng XW, Geng L, Zheng SS (2015). Reversible sinusoidal obstruction syndrome associated with tacrolimus following liver transplantation. World J Gastroenterology.

[17] Mori T, Shimizu T, Yamazaki R (2005). Altered metabolism of tacrolimus in hepatic veno-occlusive disease. Transplant international.

[18] Prashar R, Stewart D, Moza A (2017). Tacrolimus as a Rare Cause of Pericardial Effusion in a Renal Transplant Recipient. Heart Views.

